# *Angiostrongylus vasorum* in Domestic Dogs in Castilla y León, Iberian Peninsula, Spain

**DOI:** 10.3390/ani11061513

**Published:** 2021-05-23

**Authors:** Rodrigo Morchón, José Alberto Montoya-Alonso, José Ángel Sánchez-Agudo, Juan de Vicente-Bengochea, Xiomara Murcia-Martínez, Elena Carretón

**Affiliations:** 1 Zoonotic Disease and One Heatlh Group, Faculty of Pharmacy, Campus Miguel Unamuno, University of Salamanca, 37007 Salamanca, Spain; juandevicente@usal.es (J.d.V.-B.); xiomaramurcia@usal.es (X.M.-M.); 2 Internal Medicine, Faculty of Veterinary Medicine, Research Institute of Biomedical and Health Sciences (IUIBS), University of Las Palmas de Gran Canaria, 35413 Las Palmas de Gran Canaria, Spain; alberto.montoya@ulpgc.es; 3 Research Group on Biodiversity, Human Diversity and Conservation Biology, Campus Miguel Unamuno, University of Salamanca, 37007 Salamanca, Spain; jasagudo@usal.es

**Keywords:** *Angiostrongylus vasorum*, Castilla y León, Spain, Europe, epidemiology, GIS

## Abstract

**Simple Summary:**

Canine angiostrongylosis is a vascular and pulmonary disease caused by *Angiostrongylus vasorum*. In Europe, there has been an increasing number of studies showing a rise in the studies in both domestic and wild canids. In Spain, angiostrongylosis is still little-known, and studies are scarce. The aim of this study was to analyze the presence of *A. vasorum* in 1475 domestic dogs from the autonomous region of Castilla y León (Spain). Antigens of this species were found in 0.75% of the tested dogs, most of which lived outdoors, a significant risk factor. The geographic information system (GIS) analysis showed that the infected animals mainly lived in areas with mild temperatures and climate during most of the year and close to water bodies: stagnant water; riverbanks or irrigated areas with a predominance of alder, holm oak and gall oak forests. One main conclusion was the need to carry out more studies in countries and areas with the presence of this parasite in order to know the prevalence in dogs and wild canids, as well as determine the environmental factors that influence its presence, to be able to take more effective measures to control this disease.

**Abstract:**

*Angiostrongylus vasorum* is the causative agent of canine angiostrongylosis, a disease affecting domestic and wild canids. In Europe, it is an emerging disease, mainly reported in red foxes. In Spain, there are a few studies that address the prevalence and pathology of this disease. Castilla y León is the largest region of the Iberian Peninsula, whose extensive area is 94,224 km^2^; however, until now, there have been no epidemiological studies on this disease. Therefore, the aim of this study was to analyze the presence of antigens of *A. vasorum* in 1475 dogs from Castilla y León, showing an overall prevalence of 0.75%. The infected dogs were mainly outdoors, guard and hunting breed dogs and living in locations with mild climates close to areas of high edaphic humidity, such as stagnant water, irrigated crops or riverbanks, with the vegetation dominated by alders, holm oak and gall oak forests, where the intermediate hosts develop. It is necessary to carry out more in-depth studies on the epidemiology and pathology of this disease in Spain and Europe in order to carry out efficient control in both domestic and wild animals.

## 1. Introduction

Angiostrongylosis is a disease caused by the metastrongyloid nematode *Angiostrongylus vasorum* (Baillet, 1866) (Nematoda: Angiostrongylidae), which affects domestic dogs and other wild carnivores—mainly foxes, although it has also been described in wolves, jackals and badgers [1,2,3,4]. Adult parasites (13–21 mm) live in the host’s pulmonary arteries and right heart chambers, where they produce eggs bound to the respiratory tract that subsequently hatch into L_1_ larvae. These larvae penetrate the pulmonary alveoli and later migrate to the oropharynx, where they are swallowed, entering the digestive system, and are finally eliminated through feces within two months of infection. These L_1_ larvae infect gastropod mollusks (snails, slugs and frogs), which act as intermediate hosts and develop the infective L_3_ stage within 10–16 days. Canids become infected by ingesting intermediate hosts, as well as contaminated grass or puddle water. L_3_ larvae enter the portal circulation through the intestinal wall and migrate to the abdominal lymph nodes and molt to L_4_. Finally, they enter the portal circulation, migrate through the liver parenchyma and, eventually, reach the right ventricle and pulmonary arteries, where they finish their development as adults, which is their final location. The prepatent period lasts between 38 and 57 days [2,5,6].

*A. vasorum* can cause severe disease in dogs, producing cardiorespiratory signs, which are the most frequent, as well as hemorrhagic diathesis, neurological signs and other nonspecific signs, although many dogs may remain asymptomatic. The symptoms are due to the presence of adult nematodes and eggs in the pulmonary vasculature, as well as to the migration of L_1_ larvae from the pulmonary capillaries to the alveolar walls and bronchial tree. Furthermore, damage to the endothelial surface of the pulmonary vasculature may lead to local activation of the coagulation and inflammatory system, causing thrombosing arteritis, which will contribute to the pathogenesis of the disease. In chronic infections, a granulomatous response in the lungs and pulmonary fibrosis contributes to the generation of clinical signs. Moreover, this disease can be fatal if not properly treated [4,7,8]. The generally nonspecific symptomatology makes diagnosing of the disease difficult, which is one of the reasons why canine angiostrongylosis is underdiagnosed [5].

Currently, angiostrongylosis is considered an emerging disease in many European countries, e.g., Germany, France, Greece, Italy, Romania, Sweden, Turkey, UK, Denmark, Portugal, Hungary, Ireland and Switzerland. The climate and vegetation of those countries is one of the most important factors for the presence of *A. vasorum,* where it has been reported mainly in red foxes, although cases in dogs are increasing considerably [9,10,11,12].

In Spain, there are very few studies addressing the epidemiology and pathology of *A. vasorum*, and most of them have reported the presence of this parasite in wild hosts such as wolves, foxes and badgers in different autonomous communities (Asturias, Aragón, Galicia, Castilla y León, Catalonia, Murcia and the Basque Country) [6,13,14,15]; moreover, there has only been one study addressing the epidemiology of the disease in dogs in Spain, with a mean prevalence of 1.73% [6]. Castilla y León is the largest region of the Iberian Peninsula; with a surface area of over 94,000 km^2^, its extension exceeds the surface of many European countries (e.g., Denmark, Portugal, Hungary, Ireland or Switzerland). The prevalence reported in a previous study was 0.93%, although only two of the nine provinces that compose Castilla y León were analyzed (Salamanca and León) [6]. On the other hand, in the surrounding regions of Castilla y León (Galicia, Cantabria, Asturias, Basque Country, Aragón and Northern Portugal), in previous studies, the prevalence reported was from 0.93% to 3% in domestic dogs, from 33.3% to 43.2% in foxes, 21.6% in wolves and 24% in the Eurasian badger [6,16,17,18,19].

The implementation of global positioning systems for the geolocation of data by using geographical information systems (GIS) has been shown to have a great capacity to understand the spatial patterns of distribution of biological events in a territory and the biotic and abiotic processes that explain them [20]. This methodology, which explicitly shows the environmental variability of a territory, together with spatial correlation analyses, allow the elaboration of predictive models that focus on the determination of the capacity of each space to host a biological event based on the predictor variables used. In this specific field of this study, this methodology has been used to calculate the potential risks of parasitic diseases either on a global scope, i.e., [21] or a local area [22,23].

Being aware that Castilla y León, one of the largest regions in Europe, with vast climatological and orographic diversity, lacks epidemiological studies, the aim of this study was focused on the analysis of the prevalence and distribution of *A. vasorum* in dogs living in this territory.

## 2. Materials and Methods

### 2.1. Study Area

**Figure 1:**
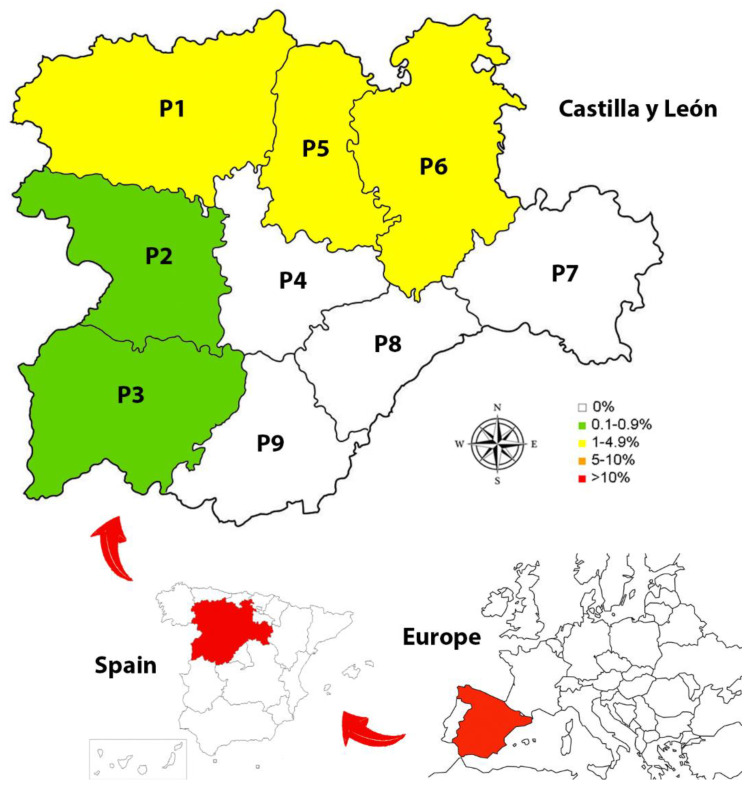
Prevalence of *Angiostrongylus vasorum* in the 9 provinces of Castilla y León, Spain: (P1) León; (P2) Zamora; (P3) Salamanca; (P4) Valladolid; (P5) Palencia; (P6) Burgos; (P7) Soria; (P8) Segovia; (P9) Ávila.

Castilla y León is an autonomous community of Spain, located in the northwestern quadrant of the Iberian Peninsula. Its extensive area of 94,224 km^2^ makes it the largest autonomous community, region or district in Europe and even exceeds seven of the fifteen-member countries of the European Union. Castilla y León is divided into 9 provinces (León, Zamora, Salamanca, Valladolid, Palencia, Burgos, Soria, Segovia and Ávila) (Figure 1). Its orography is mainly characterized by a plateau, with an average altitude of around 800 m above sea level, bounded by the Cantabrian Mountains to the north, the Montes de León to the northwest, the Cordillera Central to the south, the Cordillera Iberica to the east and the border with Portugal to the west. The main river is the Duero, which crosses the community from east to west, configuring wide river valleys that generate some deep ravines [24,25].

According to Köppen’s climate classification, Castilla y León presents a Continental Mediterranean climate, with an average annual rainfall of 450–500 mm, cold winters (average temperatures between 3–6 °C in January) and short, hot summers (average temperatures of 19 to 22 °C) in which the aridity of the Mediterranean climate is evident. Different sub-climates are described in the territory: Csb (temperate with dry or temperate summer) or Cfb (temperate with a dry season and temperate summer) sub-climates, both characterized by an average temperature of the warmest month below 22 °C but above 10 °C for five or more months per year. In several areas of the central plateau, the sub-climate is classified as Csa (temperate with a dry or hot summer), as it exceeds 22 °C during the summer, and as Bsk (cold steppe), where the average annual temperature is below 18 °C. At high altitudes in the Cantabrian Mountains and other mountain areas, there is a cold, temperate climate with average temperatures below 3 °C in the coldest months and dry summers (Dsb or Dsc) [26]. 

According to the vegetation and the map of phytoclimatic series by Rivas Martinez [27], Castilla y León is mainly included in the Mediterranean region, supra-Mediterranean floor and Carpetano–Iberico–Leonesa biogeographic province. The potential forest formations of quercines are predominant: holm oaks (*Quercus ilex* subsp. *ballota*), pyrenean oaks (*Q. pyrenaica*), cork oaks (*Q. suber*) and Quejico oaks (*Q. faginea*), which are planted, managed and regularly pruned, configuring a manmade ecosystem characterized by a savannah-like physiognomy and locally known as “dehesa systems”. These forests cover most of the plains and middle slopes, but some beech (*Fagus sylvatica*) and chestnut (*Castanea sativa*) forests are also present in the mountainous foothills. Sabinas and other junipers (*Juniperus* sp.) practically complete most of this forest landscape, together with the riverside communities associated with the main rivers and streams that cross the territory. 

### 2.2. Sample Collection 

From September 2019 to December 2020, a total of 1475 blood samples from domestic dogs coming from all the provinces of Castilla y León were collected for the study (Table 1 and Table 2). For this, 44 veterinary clinics voluntarily collaborated by providing the samples; these were randomly collected from the canine patients who attended their clinics, provided they met the inclusion criteria. Of these, 222 came from León, 109 from Zamora, 137 from Salamanca, 166 from Valladolid, 112 from Palencia, 284 from Burgos, 118 from Soria, 224 from Segovia and 103 from Ávila. Age at presentation to the clinics, breed, sex and habitat were recorded for each dog. The criteria for inclusion were: (a) no previous history of infection by angiostrongylosis, (b) not receiving prophylactic treatment against *A. vasorum* regularly and (c) owner consent to participate in the survey. 

Blood samples were collected from the cephalic or jugular vein, placed in 3-mL serum tubes and centrifuged. Serum samples were kept at −20 °C until tests were performed. All samples were tested for the presence of circulating antigens of *A. vasorum* using Angio Detect^TM^ (IDEXX Laboratories Inc., Westbrook, ME, USA). The procedure was carried out following the manufacturer’s instructions.

### 2.3. Statistical Analysis

Data were analyzed using GraphPad Prism 8.4 (GraphPad, San Diego, CA, USA). Descriptive analysis of the variables considered was carried out considering the proportions of the qualitative variables. Chi-square and Fisher’s exact tests to compare the proportions were performed. Confidence interval (95% CI) values were also calculated. In all cases, the significance level was established at *p* < 0.05.

### 2.4. Geographic Information System (GIS) Mapping

A map of the sampling area was constructed using ArcMap v.10.8 (ESRI, 2020 Redlands, CA, USA), including the following layers of environmental information that have been considered relevant for the dynamics of the analyzed organisms and their transmission vectors: climate [27], potential vegetation [28], rivers, lakes, lagoons, irrigated croplands and parks [29]. Thematic symbols were added for easier interpretation of the map. The canine samples were georeferenced by the location of veterinary clinics where the medical consultations occurred. Therefore, the points shown on the maps correspond to the centroids of the polygons of the postal codes where the analyses of the dogs were carried out. Inferences were drawn from a proximity analysis between the presence points and the environmental characteristics of the layers used. The coordinate system used was gcs_ETRS_1989.

## 3. Results

Of the studied dogs, 49.3% were females and 50.7% were males. The dogs were classified as indoor (46%), outdoor (43.8%) or indoor/outdoor (at least 1–50% of the time spent outdoors) (10.2%). The age range of the dogs that participated in the study was from 4 months to 17 years; the dogs were further divided into five age groups (Table 1). When breed was assessed, 27.2% were mongrel dogs and 72.8% were purebred dogs, represented by 111 breeds. 

The overall prevalence of canine angiostrongylosis in the studied areas of Castilla y León was 0.75% (CI: 0.42–1.33%). The prevalence was similar between females (0.83%) (CI: 0.38–1.79%) and males (0.67%) (CI: 0.29–1.56%), with no statistically significant differences between them. No positives were found in any of the dogs < 1 year and > 15 years. The highest overall prevalence was recorded in the group of 5.1–10 years (0.92%) (CI: 0.39–2.14%), followed by the dogs between 10.1–15 years (0.72%) (CI: 0.20–2.58%) and between 1–5 years (0.71%) (CI: 0.28–1.81%); no significant differences were found between the age groups. Significant differences were demonstrated between indoor and outdoor dogs (*p* < 0.05) and between indoor and indoor/outdoor dogs (*p* < 0.05).

By breed, no significant differences were found between the prevalence obtained in mongrel dogs (0.5%) (CI: 0.14–1.80%) and purebred dogs (0.84%) (CI: 0.44–1.58%). Purebred dogs were grouped as company dogs or working dogs (guard, hunting or herding). The most-represented breeds among company dogs were Yorkshire Terrier (25.3%), Maltese (6.9%) and French Bulldog (5.5%), while the most represented breeds among the working dogs were German Shepherd (11.4%), Labrador Retriever (9.8%), Spanish Hound (7%) and Boxers (6.6%). Among the company dogs (*n* = 419), the prevalence obtained was 0.24% (CI: 0.04–1.34%), while among the working dogs (*n* = 655), the prevalence was 1.22% (CI: 0.62–2.39%) (*p* < 0.05).

Of the provinces analyzed, the highest prevalence was shown in Palencia (1.79%) (CI: 0.49–6.28%), followed by León (1.80%) (CI: 0.70–4.54%), Burgos (1.06%) (CI: 0.36–3.06%), Zamora (0.92%) (CI: 0.16–5.01%) and Salamanca (0.73%) (CI: 0.13–4.02%) (Table 2). No infected animals were found in the other provinces. 

**Figure 2:**
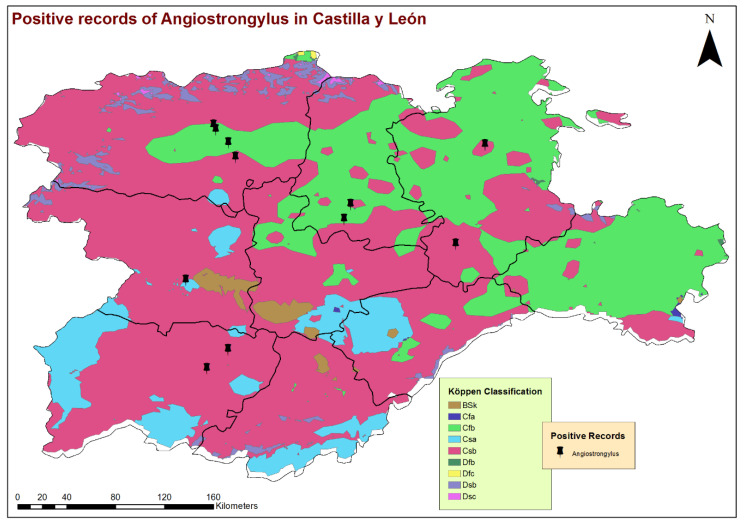
Geolocation of the dogs infected by *Angiostrongylus vasorum* and the different climatologies according to Köppen’s climate classification.

**Figure 3:**
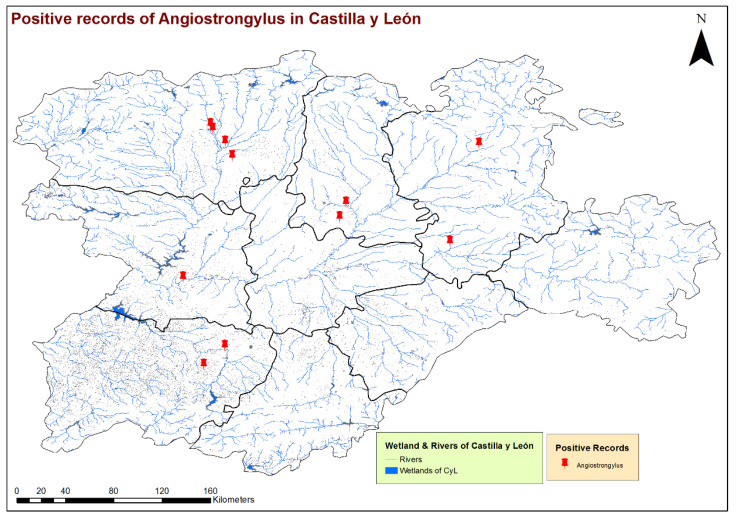
Location of the wetlands and rivers and geolocation of the dogs infected by *Angiostrongylus vasorum* in the 9 provinces of Castilla y León, Spain.

**Figure 4:**
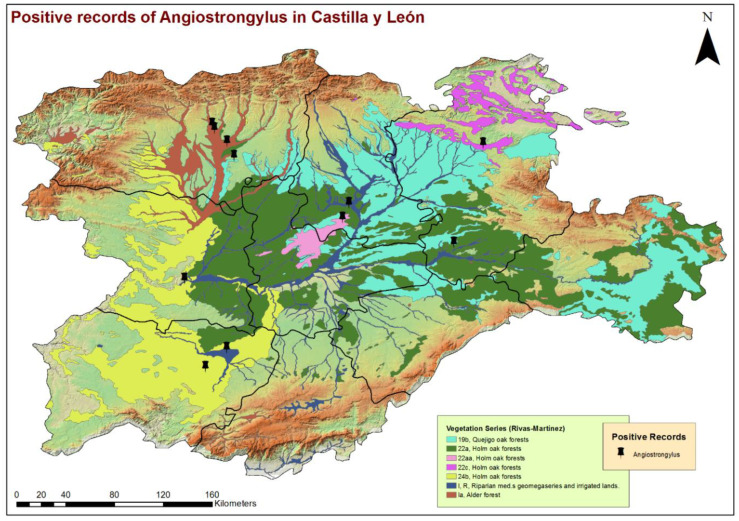
Location of the phytoclimatic series and geolocation of the dogs infected by *Angiostrongylus vasorum* in the 9 provinces of Castilla y León, Spain.

By the eco-epidemiological areas, the location of infected dogs and highest estimates of prevalence corresponded mostly with the Cfb and Csb sub-climates (Figure 2), which, in the bioclimatic context of the region, indicate a trend towards a less seasonal character with an oceanic influence, moderate temperatures and greater precipitation. All infected dogs were located in areas with high concentrations of water bodies or high soil humidity, such as stagnant water, irrigated lands or riverbanks (Figure 3). These previous bioclimatic circumstances are largely responsible for the predominant vegetation types in the vicinity of the positive cases having certain hydrophilic characteristics, as is the case of alder forests (*Alnus glutinosa*) or Quejigo oak forests, especially in the north, and riparian forests or irrigated lands in the south. Even the positive cases located in the central zone and linked to forest formations somewhat “drier” (holm oak forest) are also close to important bodies of water (Figure 4).

## 4. Discussion

Angiostrongylosis is an emerging disease in many European countries. In those regions where infections have been reported in wild animals, its presence has been demonstrated in domestic dogs as well [6,17], and, in recent years, many studies have been carried out regarding the presence of *A. vasorum* in domestic dogs in Europe [5,6,30,31,32,33,34]. The results have varied widely in different studies; one of the reasons could be the diversity in the diagnostic methods used, which varied from the detection of post-mortem adult worms in the pulmonary artery and right ventricle, serological tests focused on the detection of circulating antigens or antibodies, to the detection of *A. vasorum* larvae by the Baermann technique, mainly because sensitivity and specificity vary between the different techniques used [6,12,19,35,36,37,38,39].

The use of serological techniques is one of the most reliable diagnostic methods implemented in recent published studies. One of the most widely used tests is Angio Detect^TM^, which detects circulating antigens with a sensitivity of 98.1% and specificity of 99.4% [6,36,37,38,39]. However, previous studies have shown that this test detects the presence of circulating antigens from 5 months post-infection, so the diagnosis of earlier infections could be limited [35,36].

In a previous study carried out in Spain, the overall prevalence was 1.73%; furthermore, the study included León and Salamanca, which showed a prevalence of 0.93% [6]. In the provinces of Castilla y León that were previously analyzed, the current results showed higher incidences, obtaining a prevalence of 1.8% in León and 0.73% in Salamanca. These differences in the results may have been due to the different sample sizes or the distribution of the dogs studied, since the infected dogs were found located in areas close to wetlands and specific forests. This is consistent with previous studies, since it has been described that the prevalence of parasites in foxes and the incidence in dogs is restricted to endemic foci and, apparently, only sporadically occur outside these foci [40].

Furthermore, León borders Galicia and Asturias, which were previously shown to have a high prevalence (1.86% and 2.74%, respectively) [6]; Zamora and Salamanca border Portugal, where previous studies showed a variable prevalence from 0.66% to 5% [12]. Palencia and Burgos border Cantabria and Basque Country to the north, which previously showed a higher prevalence (2.74%), possibly due to the climate present in those regions [6].

There are few data on the distribution of this parasite among the wild fauna of Castilla y León, only reported in León [41]. However, in the autonomous communities that surround the studied area, high incidences have been reported in wolves and, especially, foxes, from 7.1% to reaching incidences of 33% and 43% [18,19,42]. These high incidences make wildlife an important reservoir of canine angiostrongylosis. Furthermore, *A. vasorum* is spreading throughout Europe and is currently considered an emerging disease, so conducting studies to determine its distribution, as well as taking control and prophylactic measures, is essential [40].

When compared to other European countries, the obtained prevalence is lower than those reported in Northern Europe and neighboring countries such as Portugal [17,43]. However, as previously mentioned, the real prevalence could be higher than that obtained in the present study, since the sensitivity of this test to detect recent infections is low [32,39], and other epidemiologic studies carried out by the detection of circulating antigens have shown a low prevalence [31,35]; in fact, studies carried out in neighboring countries (i.e., Portugal and France) using the same methodology have shown a similar prevalence, lower than that observed in the present study [12,44]. Therefore, the comparison between the prevalence obtained in different studies is not possible, due to the variability in the sensitivity and specificity of the different diagnostic methods used in each of the studies.

No significant differences were found in the results regarding sex or age, contrary to a previous study that showed significantly a higher prevalence in younger dogs [6]. As previously stated, it could be caused by the differences in sampling and distribution of the animals studied. Regarding sex, the results are similar to previous studies, which concluded that this was not a risk factor for infection. On the other hand, the significant differences found between indoor dogs when compared to indoor/outdoor and outdoor dogs were similar to other vector-borne diseases; clearly, an increased exposure to the vector increases the risk of infection [45,46,47]. The prevalence was higher in guard and hunting breed dogs; precisely, these dogs spend more hours in outdoor environments, which increases the opportunity to ingest intermediate hosts.

The GIS analysis made it possible to georeference positive cases in the geographical context of the study, which is an important starting point to develop other types of studies that serve to determine the environmental variables that contribute to the presence and spread of this disease. The layers used as a model for this first analysis, climatic, hydric and vegetation, indicate a relationship of the parasite or its vectors, with a relatively high presence of water in the substrate. Therefore, all infected animals were located in climates with mild, average temperatures and few dry seasons, close to areas with high humidity or access to water and mostly forests with the main presence of alders, Quejigo and holm oaks. These biotic and abiotic characteristics, as is known, favor the establishment and development of intermediate hosts and, thus, the transmission of the disease [48,49].

## 5. Conclusions

Angiostrongylosis is an emerging disease in Europe and, in particular, in Spain, so prevention and control measures are important. Castilla y León covers a large territory; it is the largest autonomous community or district in Europe, even larger than some European countries. Therefore, epidemiological studies such as this one are necessary, especially to raise awareness of this disease among dog owners and clinical veterinarians. Likewise, more studies are needed to complete the epidemiological map of the disease in Europe and to carry out follow-ups in areas where the disease is present.

## Figures and Tables

**Table 1:** Numbers of dogs sampled and prevalence of *Angiostrongylus vasorum* obtained by sex, age groups and habitat in Castilla y León, Spain: (*n*) number of dogs sampled; (+) positive animals; (%) percentage of positive animals; CI (95%) confidence interval.

	*n*	+	%	CI (95%)
SEX				
Male	748	5	0.67	0.29–1.56
Female	727	6	0.83	0.38–1.79
AGE				
<1 year	72	0	0.00	0–5.06
1–5 years	564	4	0.71	0.28–1.81
5.1–10 years	543	5	0.92	0.39–2.14
10.1–15 years	279	2	0.72	0.2–2.58
>15 years	17	0	0.00	0.18.43
HABITAT				
Indoor	678	1	0.15	0.03–0.83
Outdoor	646	8	1.24	0.63–2.42
Indoor/Outdoor	151	2	1.32	0.36–4.7
TOTAL	1475	11	0.75	0.42–1.33

**Table 2:** Numbers of dogs sampled and prevalence of *Angiostrongylus vasorum* by sex, age and habitat in the 9 provinces of Castilla y León, Spain. (*n*) number of dogs sampled; (+) positive animals; (%) percentage of positive animals; (CI) 95% confidence interval.

Provinces	León			Zamora			Salamanca			Valladolid			Palencia			Burgos			Soria			Segovia			Ávila		
	+	*n*	% (CI)	+	*n*	% (CI)	+	*n*	% (CI)	+	*n*	% (CI)	+	*n*	% (CI)	+	*n*	% (CI)	+	*n*	% (CI)	+	*n*	% (CI)	+	*n*	% (CI)
SEX																											
Male	2	121	1.65 (0.45–5.83)	1	65	1.54 (0.27–8.21)	0	68	0.00 (0.00–5.35)	0	88	0.00 (0.00–4.18)	1	50	2.00 (0.35–10.50)	1	128	0.78 (0.14–4.29)	0	61	0.00 (0.00–5.92)	0	110	0.00 (0.00–3.37)	0	57	0.00 (0.00–6.31)
Female	2	101	1.98 (0.54–6.93)	0	44	0.00 (0.00–8.03)	1	69	1.45 (0.26–7.76)	0	78	0.00 (0.00–4.69)	1	62	1.61 (0.29–8.59)	2	156	1.28 (0.35–4.55)	0	57	0.00 (0.00–6.31)	0	114	0.00 (0.00–3.26)	0	46	0.00 (0.00–7.71)
AGE																											
<1 year	0	8	0.00 (0.00–32.44)	0	8	0.00 (0.00–32.44)	0	9	0.00 (0.00–29.91)	0	5	0.00 (0.00–43.45)	0	2	0.00 (0.00–65.76)	0	22	0.00 (0.00–14.86)	0	6	0.00 (0.00–39.03)	0	8	0.00 (0.00–32.44)	0	4	0.00 (0.00–48.99)
1–5 years	2	64	3.13 (0.86–10.70)	1	48	2.08 (0.37–10.90)	0	41	0.00 (0.00–8.57)	0	63	0.00 (0.00–5.75)	0	40	0.00 (0.00–8.76)	1	97	1.03 (0.18–5.61)	0	65	0.00 (0.00–5.58)	0	88	0.00 (0.00–4.18)	0	58	0.00 (0.00–6.21)
5.1–10 years	1	82	1.22 (0.22–6.59)	0	37	0.00 (0.00–9.41)	1	65	1.54 (0.27–8.21)	0	55	0.00 (0.00–6.53)	2	43	4.65 (1.28–15.46)	1	105	0.95 (0.17–5.20)	0	35	0.00 (0.00–9.89)	0	85	0.00 (0.00–4.32)	0	36	0.00 (0.00–9.64)
10.1–15 years	1	62	1.61 (0.29–8.59)	0	16	0.00 (0.00–19.36)	0	21	0.00 (0.00–15.46)	0	43	0.00 (0.00–8.20)	0	25	0.00 (0.00–13.32)	1	53	1.89 (0.33–9.94)	0	11	0.00 (0.00–25.88)	0	43	0.00 (0.00–8.20)	0	5	0.00 (0.00–43.45)
>15 years	0	6	0.00 (0.00–39.03)	0	0	0.00	0	1	0.00 (0.00–79.34)	0	0	0.00	0	2	0.00 (0.00–65.76)	0	7	0.00 (0.00–35.43)	0	1	0.00 (0.00–79.34)	0	0	0.00	0	0	0.00
HABITAT																											
Indoor	1	154	0.65 (0.11–3.59)	0	63	0.00 (0.00–5.75)	0	23	0.00 (0.00–14.31)	0	83	0.00 (0.00–4.42)	0	65	0.00 (0.00–5.58)	0	116	0.00 (0.00–3.21)	0	21	0.00 (0.00–15.46)	0	122	0.00 (0.00–3.05)	0	31	0.00 (0.00–11.02)
Outdoor	3	65	4.62 (1.58–12.71)	1	46	2.17 (0.38–11.33)	1	114	0.88 0.16–4.80)	0	83	0.00 (0.00–4.42)	1	21	4.76 (0.85–22.67)	2	99	2.02 (0.56–7.07)	0	83	0.00 (0.00–4.42)	0	63	0.00 (0.00–5.75)	0	72	0.00 (0.00–5.06)
Indoor/Outdoor	0	3	0.00 (0.00–56.15)	0	0	0.00	0	0	0.00	0	0	0.00	1	26	3.85 (0.68–18.89)	1	69	1.45 (0.26–7.76)	0	14	0.00 (0.00–21.53)	0	39	0.00 (0.00–8.97)	0	0	0.00
TOTAL	4	222	1.80 (0.70–4.54)	1	109	0.92 (0.16–5.01)	1	137	0.73 (0.13–4.02)	0	166	0.00 (0.00–2.26)	2	112	1.79 (0.49–6.28)	3	284	1.06 (0.36–3.06)	0	118	0.00 (0.00–3.15)	0	224	0.00 (0.00–1.69)	0	103	0.00 (0.00–3.60)

## Data Availability

The study did not report any data.

## References

[1] Helm J.R., Morgan E.R., Jackson M.W., Wotton P., Bell R. (2010). Canine angiostrongylosis: An emerging disease in Europe. J. Vet. Emerg. Crit. Care (San Antonio).

[2] Elsheikha H.M., Holmes S.A., Wright I., Morgan E.R., Lacher D.W. (2014). Recent advances in the epidemiology, clinical and diagnostic features, and control of canine cardio-pulmonary angiostrongylosis. Vet. Res..

[3] Lemming L., Jørgensen A.C., Nielsen L.B., Nielsen S.T., Mejer H., Chriél M., Petersen H.H. (2020). Cardiopulmonary nematodes of wild carnivores from Denmark: Do they serve as reservoir hosts for infections in domestic animals?. Int. J. Parasitol. Parasites Wildl..

[4] Paradies P., Sasanelli M., Capogna A., Mercadante A., Rubino G.T.R., Bussadori C.M. (2021). Is Pulmonary Hypertension a Rare Condition Associated to Angiostrongylosis in Naturally Infected Dogs?. Top. Companion Anim. Med..

[5] Morgan E.R., Jefferies R., van Otterdijk L., McEniry R.B., Allen F., Bakewell M., Shaw S.E. (2010). *Angiostrongylus vasorum* infection in dogs: Presentation and risk factors. Vet. Parasitol..

[6] Carretón E., Morchón R., Falcón-Cordón Y., Matos J., Costa-Rodríguez N., Montoya-Alonso J.A. (2020). First epidemiological survey of *Angiostrongylus vasorum* in domestic dogs from Spain. Parasit. Vectors.

[7] Bagardi M., Rabbogliatti V., Bassi J., Gioeni D., Oltolina M., Villa L. (2021). *Angiostrongylus vasorum* in a Red Panda (*Ailurus fulgens*): Clinical Diagnostic Trial and Treatment Protocol. Acta Parasitol..

[8] De Zan G., Citterio C.V., Danesi P., Gaspardis G., Gabassi E., Panciera L., Zanardello C., Binato G., Cocchi M. (2021). Angiostrongylosis in northeastern Italy: First report of two autochthonous fatal cases in dogs and first detection in a wild red fox. Vet. Parasitol. Reg. Stud. Rep..

[9] Saeed I., Maddox-Hyttel C., Monrad J., Kapel C.M. (2006). Helminths of red foxes (*Vulpes vulpes*) in Denmark. Vet Parasitol..

[10] Tachmazidou A., Papaioannou N., Diakou A., Savvas I., Patsikas M., Stylianaki I., Morelli S., Di Cesare A., Mylonakis M.E. (2021). First report of fatal autochthonous angiostrongylosis in a dog in Greece. Vet. Parasitol. Reg. Stud. Rep..

[11] Al-Sabi M.N., Halasa T., Kapel C.M. (2014). Infections with cardiopulmonary and intestinal helminths and sarcoptic mange in red foxes from two different localities in Denmark. Acta Parasitol..

[12] Alho A.M., Schnyder M., Schaper R., Meireles J., Belo S., Deplazes P., de Carvalho L.M. (2016). Seroprevalence of circulating *Angiostrongylus vasorum* antigen and parasite-specific antibodies in dogs from Portugal. Parasitol. Res..

[13] Gotázar C., Villafuerte R., Lucientes J., Fernández-de-Luco D. (1998). Habitat related differences in helminth parasites of red foxes in the Ebro valley. Vet. Parasitol..

[14] Mañas S., Ferrer D., Castellà J., Maria López-Martín J. (2005). Cardiopulmonary helminth parasites of red foxes (*Vulpes vulpes*) in Catalonia, northeastern Spain. Vet. J..

[15] Martínez-Carrasco C., Ruiz De Ybáñez M.R., Sagarminaga J.L., Garijo M.M., Moreno F., Acosta I., Hernández S., Alonso F.D. (2007). Parasites of the red fox (*Vulpes vulpes* Linnaeus, 1758) in Murcia, southeast Spain. Revue Méd. Vét..

[16] Álvarez F., Iglesias R., Bos J., Rey J., Sanmartin Durán M.L. (1991). Lung and hearth nematodes in some Spanish mammals. Wiad. Parazytol..

[17] Alho A.M., Meireles J., Schnyder M., Cardoso L., Belo S., Deplazes P., de Carvalho L.M. (2018). *Dirofilaria immitis* and *Angiostrongylus vasorum*: The current situation of two major canine heartworms in Portugal. Vet. Parasitol..

[18] Martínez-Rondán F.J., Ruiz de Ybáñez M.R., López-Beceiro A.M., Fidalgo L.E., Berriatua E., Lahat L., Sacristán I., Oleaga Á., Martínez-Carrasco C. (2019). Cardiopulmonary nematode infections in wild canids: Does the key lie on host-prey-parasite evolution?. Res. Vet. Sci..

[19] Gerrikagoitia X., Barral M., Juste R.A. (2010). *Angiostrongylus* species in wild carnivores in the Iberian Peninsula. Vet. Parasitol..

[20] Pullan R.L., Sturrock H.J.W., Soares Magalhaes R.J., Clements A.C.A., Brooker S.K. (2012). Spatial parasite ecology and epidemiology: A review of methods and applications. Parasitology.

[21] Weiss D.J., Lucas T.C.D., Nguyen M., Nandi A.K., Bisanzio D., Battle K.E., Cameron E., Twohig K.A., Pfeffer D.A., Rozier J.A. (2019). Mapping the global prevalence, incidence, and mortality of *Plasmodium falciparum*, 2000–17: A spatial and temporal modelling study. Lancet.

[22] Sifaki-Pistola D., Ntais P., Christodoulou V., Mazeris A., Antoniou M. (2014). The Use of Spatial Analysis to Estimate the Prevalence of Canine Leishmaniasis in Greece and Cyprus to Predict Its Future Variation and Relate It to Human Disease. Am. J. Trop. Med. Hyg..

[23] Rinaldi L., Biggeri A., Carbone S., Musella V., Catelan D., Veneziano V., Cringoli G. (2006). Canine faecal contamination and parasitic risk in the city of Naples (southern Italy). BMC Vet. Res..

[24] (2021). European Commision. https://ec.europa.eu/.

[25] (2021). Research Innovation. https://fuescyl.com/images/03innovacion_conocimiento/Comisionado/RIS3_Castilla_y_Leon_2014-2020_(eng).pdf.

[26] Agencia Estatal de Meteorología, Ministerio de Agricultura, Alimentación y Medio Ambiente. In: Iberian Climate Atlas. Air Temperature and Precipitation (1971–2000). https://www.aemet.es/documentos/es/conocermas/publicaciones/Atlas-climatologico/Atlas.pdf.

[27] Köppen’s Climate Classification. http://koeppen-geiger.vu-wien.ac.at/shifts.htm.

[28] Rivas Martínez S. (1987). Memoria del Mapa de Series de Vegetación de España 1: 400.000. 268 pp. ICONA. Ministerio de Agricultura. https://www.miteco.gob.es/es/biodiversidad/.

[29] Centro Nacional de Información Geográfica. http://centrodedescargas.cnig.es/CentroDescargas.

[30] Bird L., Bilbrough G., Fitzgerald R. (2014). Controlling *Angiostrongylus vasorum* infection in dogs. Vet. Record.

[31] Deak G., Gillis-Germitsch N., Ionică A.M., Mara A., Păstrav I.R., Cazan C.D., Ioniță M., Mitrea I.L., Răileanu C., Bărburaș D. (2019). The first seroepidemiological survey for *Angiostrongylus vasorum* in domestic dogs from Romania. Parasit. Vectors.

[32] Lempereur L., Martinelle L., Marechal F., Bayrou C., Dalemans A.C., Schnyder M., Losson B. (2016). Prevalence of *Angiostrongylus vasorum* in southern Belgium, a coprological and serological survey. Parasit. Vectors.

[33] Penagos-Tabares F., Lange M.K., Chaparro-Gutiérrez J.J., Taubert A., Hermosilla C. (2018). *Angiostrongylus vasorum* and *Aelurostrongylus abstrusus*: Neglected and underestimated parasites in South America. Parasit. Vectors.

[34] Tiškina V., Lindqvist E.L., Blomqvist A.C., Orav M., Stensvold C.R., Jokelainen P. (2019). Autochthonous *Angiostrongylus vasorum* in Finland. Vet. Rec. Open.

[35] Schnyder M., Schaper R., Pantchev N., Kowalska D., Szwedko A., Deplazes P. (2013). Serological detection of circulating *Angiostrongylus vasorum* antigen- and parasite-specific antibodies in dogs from Poland. Parasitol. Res..

[36] Schnyder M., Stebler K., Naucke T.J., Lorentz S., Deplazes P. (2014). Evaluation of a rapid device for serological in-clinic diagnosis of canine angiostrongylosis. Parasit. Vectors.

[37] Schnyder M., Jefferies R., Schucan A., Morgan E.R., Deplazes P. (2015). Comparison of coprological, immunological and molecular methods for the detection of dogs infected with *Angiostrongylus vasorum* before and after anthelmintic treatment. Parasitology.

[38] Schnyder M., Maurelli M.P., Morgoglione M.E., Kohler L., Deplazes P., Torgerson P., Cringoli G., Rinaldi L. (2011). Comparison of faecal techniques including FLOTAC for copromicroscopic detection of first stage larvae of *Angiostrongylus vasorum*. Parasitol. Res..

[39] Liu J., Schnyder M., Willesen J.L., Potter A., Chandrashekar R. (2017). Performance of the Angio Detect™ in-clinic test kit for detection of *Angiostrongylus vasorum* infection in dog samples from Europe. Vet. Parasitol. Reg. Stud. Rep..

[40] Morgan E.R., Jefferies R., Krajewski M., Ward P., Shaw S.E. (2009). Canine pulmonary angiostrongylosis: The influence of climate on parasite distribution. Parasitol. Int..

[41] Segovia J.M., Torres J., Miquel J. (2004). Helmith parasites of the red fox (*Vulpes vulpes* L., 1758) in the Iberan Peninsula: An ecological study. Acta Parasitol..

[42] Figueiredo A., Oliveira L., Madeira de Carvalho L., Fonseca C., Torres R.T. (2016). Parasite species of the endangered Iberian wolf (*Canis lupus signatus*) and a sympatric widespread carnivore. Int. J. Parasitol. Parasites Wildl..

[43] Majoros G., Fukár O., Farkas R. (2010). Autochtonous infection of dogs and slugs with *Angiostrongylus vasorum* in Hungary. Vet. Parasitol..

[44] Schnyder M., Bilbrough G., Hafner C., Schaper R. (2017). *Angiostrongylus vasorum*, “The French Heartworm”: A Serological Survey in Dogs from France Introduced by a Brief Historical Review. Parasitol. Res..

[45] Franklinos L.H.V., Jones K.E., Redding D.W., Abubakar I. (2019). The effect of global change on mosquito-borne disease. Lancet Infect. Dis..

[46] Montoya-Alonso J.A., Morchón R., Costa-Rodríguez N., Matos J.I., Falcón-Cordón Y., Carretón E. (2020). Current Distribution of Selected Vector-Borne Diseases in Dogs in Spain. Front. Vet. Sci..

[47] Roussel. C., Drake J., Ariza J.M. (2019). French national survey of dog and cat owners on the deworming behaviour and lifestyle of pets associated with the risk of endoparasites. Parasit. Vectors.

[48] Čabanová V., Miterpáková M., Druga M., Hurníková Z., Valentová D. (2018). GIS-based environmental analysis of fox and canine lungworm distribution: An epidemiological study of *Angiostrongylus vasorum* and *Crenosoma vulpis* in red foxes from Slovakia. Parasitol. Res..

[49] Maksimov P., Hermosilla C., Taubert A., Staubach C., Sauter-Louis C., Conraths F.J., Vrhovec M.G., Pantchev N. (2017). GIS-supported epidemiological analysis on canine *Angiostrongylus vasorum* and *Crenosoma vulpis* infections in Germany. Parasite. Vectors.

